# Characterization and Genomic Analysis of the Naphthalene-Degrading *Delftia tsuruhatensis* ULwDis3 Isolated from Seawater

**DOI:** 10.3390/microorganisms11041092

**Published:** 2023-04-21

**Authors:** Olesya I. Sazonova, Anastasia A. Ivanova, Yanina A. Delegan, Rostislav A. Streletskii, Diana D. Vershinina, Sergei L. Sokolov, Anna A. Vetrova

**Affiliations:** 1 Federal Research Center “Pushchino Scientific Center for Biological Research of the Russian Academy of Sciences”, 142290 Pushchino, Russia; mewgia@ya.ru (Y.A.D.); dina-20002001@mail.ru (D.D.V.); sls@ibpm.pushchino.ru (S.L.S.); phdvetrova@gmail.com (A.A.V.); 2 State Research Center for Applied Microbiology and Biotechnology, 142279 Obolensk, Russia; 3 Laboratory of Ecological Soil Science, Faculty of Soil Science, Lomonosov Moscow State University, 119991 Moscow, Russia; streletskiyrostislav@mail.ru; 4 Federal State Budgetary Educational Institution of Higher Education Pushchino State Natural Science Institute, 142290 Pushchino, Russia

**Keywords:** *Delftia tsuruhatensis*, genome, naphthalene biodegradation, *nag* operon

## Abstract

Strains of the genus *Delftia* are poorly studied microorganisms. In this work, the complete genome of the naphthalene-degrading *Delftia tsuruhatensis* strain ULwDis3 isolated from seawater of the Gulf of Finland of the Baltic Sea was assembled. For the first time, genes encoding naphthalene cleavage pathways via salicylate and gentisate were identified in a strain of the genus *Delftia*. The genes are part of one operon (*nag* genes). Three open reading frames (ORFs) were found in the genome of *D. tsuruhatensis* strain ULwDis3 that encode gentisate 1.2-dioxygenase. One of the ORFs is part of the *nag* operon. The physiological and biochemical characteristics of the strain ULwDis3 when cultured in mineral medium with naphthalene as the sole source of carbon and energy were also studied. It was found that after 22 h of growth, the strain stopped consuming naphthalene, and at the same time, naphthalene 1.2-dioxygenase and salicylate 5-hydroxylase activities were not detected. Later, a decrease in the number of living cells and the death of the culture were observed. Gentisate 1.2-dioxygenase activity was detected from the time of gentisate formation until culture death.

## 1. Introduction

Polycyclic aromatic hydrocarbons (PAHs) are highly toxic, cancerogenic, and mutagenic compounds for living organisms [1]. PAHs are ubiquitous pollutants that enter the environment not only through expanding anthropogenic activity but also through natural processes (e.g.; volcanic activity and forest fires). These compounds certainly impact the structure and functioning of the natural microbiota. This leads, on the one hand, to elimination of species lacking adaptive potential and, on the other hand, to domination of microbial community representatives with different mechanisms of pollutant degradation [2]. Naphthalene is often used as a model pollutant in laboratory experiments to study the degradation of PAHs by microorganisms. Bacteria of genera *Pseudomonas, Vibrio, Mycobacterium, Marinobacter, Sphingomonas,* and *Micrococcus* are widely known for their ability to oxidize naphthalene [3].

Metabolic versatility is a characteristic feature of the heterotrophic bacteria of the genus *Delftia*. These bacteria are strictly aerobic and oxidase-positive and do not ferment glucose. The bacterial cells of *Delftia* are motile, slightly curved or straight Gram-negative rods. The genus *Delftia* belongs to the family *Comamonadaceae* in the order *Burkholderiales* of the class *Betaproteobacteria*. This genus was described in 1999 after the reclassification of *Comamonas acidovorans* [4]. This is a group that contains only six characterized species: *D. acidovorans*, *D. tsuruhatensis*, *D. lacustris*, *D. litopenaei*, *D. rhizosphaerae*, and *D. deserti*. It is known that bacteria of this genus have a wide geographical distribution in marine and freshwater environments, rhizosphere, soil, plants, and clinical specimens. Some members of the genus *Delftia* are capable of decomposing various pollutants such as chlorobenzene, dimethylphenols, and diesel fuel [4]. However, there are few studies on the mechanisms of oil hydrocarbon degradation, including PAHs, by various strains of *Delftia* [5].

In recent years, knowledge of the mechanisms of the degradation of various hydrocarbons by bacteria has expanded due to the development of modern omics approaches. To date, the genome sequences of 90 strains of the genus *Delftia* have been published in the NCBI database. The approximate genome size ranges from 5.3 to 7.4 Mb, and the average GC content is 66%. It should be noted that the assembly level to the complete genome was detected only for 14 strains identified to the species level of *Delftia*. Genomic characterization of these bacteria revealed genetic elements responsible for the degradation of various pollutants; the synthesis of phytohormones, siderophores, and antibiotics; as well as determinants of resistance to heavy metals [4].

The complete genome sequence was determined for *Delftia acidovorans* strain Cs1–4 [6]. The Cs1–4 was isolated from PAH-contaminated soil and had the ability to degrade phenanthrene as the sole source of carbon. The article demonstrated that the catabolic genes were localized on a 232 Kb genomic island (*phn* island). The genomic island was restricted to mobile genetic elements. It was shown that the other biodegradation pathways reconstructed from the *Delftia acidovorans* Cs1–4 genome sequence included benzoate (via acetyl-CoA pathway), styrene, nicotinic acid (via the maleamate pathway), and pesticides.

Wu et al. [7] isolated a strain of *Delftia lacustris* LZ-C capable of growth on naphthalene. The authors observed the absence of one of the key naphthalene degradation genes, *nahA*. It was suggested that a new protein with a function similar to that of *nahA* (*orf05738_1*) was present in the genome of the *Delftia lacustris* LZ-C strain. The amino acid composition of the identified dioxygenase was 32% similar to the α-subunits of naphthalene 1.2-dioxygenase, NahAc. The authors showed that when 2-methylnaphthalene was degraded by the LZ-C strain, there was a 2.2-fold increase in the *orf05738_1* gene mRNA level. However, the aim of this article was not to clarify the pathway of naphthalene degradation by *Delftia lacustris* LZ-C. 

Thus, despite the fact that the genus *Delftia* has recently been actively studied, the genomic characteristics of members of the genus capable of PAH degradation have not yet been comprehensively investigated. Previously [8], strains of the genus *Delftia* capable of degrading naphthalene were studied. It was shown that these strains contained sequences of genes encoding large subunits of naphthalene 1.2-dioxygenase and salicylate 5-hydroxylase. The assumption was made that these strains degrade naphthalene through gentisate rather than catechol (Figure S1). One of these strains was chosen as an object for the present study. 

The aim of this work was to study the genomic, physiological, and biochemical features of the naphthalene-degrading strain *Delftia* sp. ULwDis3 in order to obtain a better understanding of various aspects of the microbial metabolism of PAHs in general and naphthalene degradation in particular.

## 2. Materials and Methods

### 2.1. Bacterial Strain

The strain *Delftia* sp. ULwDis3 was isolated from seawater in the Gulf of Finland of the Baltic Sea sampled near the port of Ust-Luga (Leningrad Oblast, Russia) [8]. To isolate the ULwDis3 strain, we used the method of direct plating of seawater samples on mineral agar medium with diesel fuel as the sole source of carbon and energy. First, 100 µL of diesel fuel was added to a sterile silicone tube, which was placed on the lid of an inverted Petri dish. Thus, the strain was grown in diesel fuel vapor. The ULwDis3 strain was stored in the collection of the Laboratory of plasmid biology (IBPM RAS (FRC “PSCBR” RAS), Pushchino, Russia). It was capable of utilizing naphthalene and diesel fuel at 28 ℃. The strain *D. tsuruhatensis* ULwDis3 was deposited in the All-Russian Collection of Microorganisms under the number VKM B-3175D.

### 2.2. Chemicals

Sigma-Aldrich (Burlington, MA, USA) reagents of high purity (>98%) were used: dichloromethane, gentisate, naphthalene, and salicylate. 

### 2.3. Genome Sequencing, Assembly and Annotation

Genomic DNA of *Delftia* sp. ULwDis3 was isolated and purified using a cetyltrimethylammonium bromide miniprep procedure [9]. To determine the genomic sequence of *D. tsuruhatensis* strain ULwDis3, genomic DNA sequencing was performed using Illumina and Oxford Nanopore technologies.

A MinION sequencer with FLO-MIN106 flow cell (Oxford Nanopore Technologies, Oxford, UK) was used for sequencing. A ligation sequencing kit (SQK-LSK109, UK) was used for library preparation. Illumina NovaSeq 6000 (San Diego, CA, USA) was used to sequence the same DNA. The KAPA HyperPlus kit (KAPA-biosystems) was used to prepare paired-end libraries. SPAdes software versions 3.15.2 (Saint Petersburg, Russia) [10] and Flye 2.9 [11] were used for hybrid assembly of Illumina and Nanopore reads, respectively. Bowtie2 version 2.3.5.1 [12] and Pilon version 1.23 [13] were used to correct Nanopore errors using Illumina data. Circularization of the ends of the replicon (chromosome) was confirmed by overlapping ends. The data were registered in the GenBank database under the following accession numbers: BioProject, PRJNA937730; BioSample, SAMN33417407; GenBank, NZ_ CP118775. 

Prokka software version 1.14.6 (Corvallis, OR, USA) [14] was used to annotate the assembled genome. Basic Local Alignment Search Tool (BLAST) (Bethesda, MD, USA) [15] was used to analyze the function of some proteins. Mauve software [16] was used to demonstrate the similarity and conserved synteny of genes between the type strains and the strain under study. The circular map was made using BV-BRC web resources [17]. 

The average nucleotide genome identity between the ULwDis3 strain and related strains was calculated using the OrthoANI algorithm [18]. DDH was calculated using the Genome-to-Genome Distance Calculator 2.1 service [19]. The phylogenetic tree was constructed by the neighbor-joining method using BV-BRC [20] (accessed on 4 March 2023). Genome sequences of *Delftia* strains required for constructing the phylogenetic tree were taken from NCBI [21] (accessed on 10 February 2023).

### 2.4. Growth Media and Conditions

The strain *Delftia* sp. ULwDis3 was grown at 28 °C on mineral medium with succinate (2 mg/mL) with either naphthalene (2 mg/mL), gentisate (1 mg/mL), or salicylate (0.3 mg/mL, 0.4 mg/mL, 0.5 mg/mL, 0.6 mg/mL, or 0.8 mg/mL) as the sole source of carbon and energy. The mineral medium contained (g/L): KH_2_PO_4_, 2.0; (NH_4_)_2_SO_4_, 2.0; MgSO_4_ × 7H_2_O, 0.125; NaCl, 0.5; FeSO_4_ × 7H_2_O, 0.002 (pH 7.5) [22].

Lysogeny broth (LB) agar medium [23] consisting of 5 g/L NaCl, 5 g/L yeast extract, 10 g/L tryptone, and 15 g/L agar (Panreac, Spain) was used to evaluate bacterial growth dynamics and obtain individual pure culture colonies.

To prepare the inoculum, cells were grown in tubes containing 10 mL of mineral medium and succinate (2% *w*/*v*). 

The culture was incubated for 20 h at 28 °C on a shaker at 180 rpm. The biomass was centrifuged for 10 min at 10,000 rpm to precipitate cells from the culture liquid. The resulting precipitate was then resuspended to 1 × 10^8^ CFU/mL and added to flasks. Thus, the initial concentration of cells in the medium did not exceed 1 × 10^7^ CFU/mL.

Substrate consumption, bacterial growth dynamics, and evaluation of enzymatic activity were performed in 750 μL Erlenmeyer flasks. The flasks contained 100 mL of mineral medium with naphthalene, salicylate, or gentisate. The flasks were cultured at 28 °C for 1–8 days at 180 rpm. All results were obtained in three independent biological replicates.

### 2.5. Enzyme Activity

First, 100 mL of culture medium from the flask was centrifuged to precipitate the cells. This precipitate was used to obtain a cell-free extract. Therefore, enzyme activity could be studied only from the middle of the exponential growth phase to obtain a sufficient amount of biomass. The precipitate was washed with 0.05 M phosphate buffer (pH 7.0) and resuspended in 5 mL of 0.02 M phosphate buffer (pH 7.5). The cell-free extract was obtained by ultrasonic disruption of the biomass using an MSE150 disintegrator for 1.5 min (3 × 30 s) at 4 °C. Cell debris was precipitated by centrifugation, and the supernatant was used to measure enzyme activity in the cell-free extract. Then, 100 μL of the cell-free extract was added to the reaction mixture to a final volume of 3.0 mL. Activities were determined at 25 °C, and the reaction was started by adding the cell-free extract on a UV-1800 spectrophotometer (Shimadzu, Kyoto, Japan). 

Activity of naphthalene 1.2-dioxygenase was determined spectrophotometrically by a decrease in NADH (A_340_, ε 6220 M^−1^ × cm^−1^) in a reaction mixture containing 0.1 mM naphthalene (alcohol solution), 0.1 mM NADH, 50 mM phosphate buffer (pH 7.5), and cell-free extract, considering endogenous NADH consumption by cell-free extract [24]. 

Salicylate 5-hydroxylase activity was determined spectrophotometrically by NADH loss (A_340_, ε 120 M^−1^ × cm^−1^) in a reaction mixture containing 0.1 mM NADH, 0.1 mM salicylate, 0.1 mM ferrous ammonium sulfate, 50 mM phosphate buffer (pH 7.5), and cell-free extract, considering the endogenous NADH consumption of cell-free extract [25].

The activity of gentisate 1.2-dioxygenase was determined by the rate of maleylpyruvate formation (A_330_, ε 10,800 M^−1^ × cm^−1^) in a reaction mixture containing cell-free extract, 100 mM potassium phosphate buffer (pH 7.4), and 0.1 mM gentisate [26]. 

The specific activity of enzymes was expressed in micromoles of substrate consumed or product formed per minute per 1 mg of total bacterial protein. Protein concentration was determined spectrophotometrically using the modified Bradford method [27].

### 2.6. Determination of Hydrocarbon Concentration

Naphthalene was extracted from the culture medium with dichloromethane (1:1, volume/volume). A gas chromatograph (Agilent 6890, Agilent Technologies, Santa Clara, CA, USA) with a flame ionization detector was used to estimate the naphthalene concentration. The chromatographic column was a DB-1 (30 m × 0.25 mm id, 0.25 μm). The temperature program of the oven was 40 °C with an increase of 15 °C/min. 

A high-performance liquid chromatograph (Agilent 1260, Agilent Technologies, USA) with a UV detector was used to estimate the concentration of salicylate and gentisate. To determine the metabolites, 1 mL of culture medium was first acidified to pH 2; then, the resulting suspension was passed through a filter with a 0.22 µm pore size. The wavelengths were as follows: gentisate, 280 nm; salicylate, 300 nm. A Synergi Hydro-RP chromatographic column (150 × 4.6 mm id, 4 μm) was used. The column thermostat temperature was 25 °C; the injected sample volume was 10 μL. Eluents: A, 5% acetonitrile: 5% 0.1% trifluoroacetic acid: 90% water; B, 5% 0.1% trifluoroacetic acid: 95% acetonitrile. Flow rate, 0.75 mL/min. Gradient elution: 0 min, 5%; 15 min, 15%; 22.5 min, 40%; 25 min, 40%; 25.5 min, 95%; 30 min, 95%.

Samples were analyzed using equipment of the Collaborative Use Center, Department of Soil Science, Lomonosov Moscow State University. Absolute calibration with analytical standards was used for quantitation. The correlation coefficient was 0.999. The validity of the results was confirmed by a one-factor analysis of variance (ANOVA), with significance set at *p* = 0.05. The samples were diluted 100-fold before the assay. All results are derived from five independent replicates.

The degree of hydrocarbon biodegradation (D) was calculated by the following formula:

D = (C_0_−C_i_)/C_0_ × 100 [%] (1)

where C_0_ is the concentration of hydrocarbon in experiment without microorganisms (abiotic control), and C_i_ is the concentration of hydrocarbon in the experiment with microorganisms after i hours of growth.

## 3. Results

### 3.1. Nucleotide Sequencing and Annotation

Sequencing and complete assembly of the *Delftia* sp. ULwDis3 genome indicate that it has a circular chromosome replicon of 6,944,081 bp (GC content: 66.52%) (Figure S2). The chromosome contains 6616 coding sequences, 15 rRNA clusters, 79 tRNAs, and 3 non-coding RNAs. The functions were assigned to 4956 coding sequences (CDSs), and 1664 CDSs were annotated as hypothetical proteins. We identified all genes for naphthalene degradation via gentisate and genes for decomposition of catechol via the *ortho*-cleavage pathway. We also found several genes of incomplete degradation pathways for toluene, xylene, benzoate, biphenyl, styrene, atrazine, 2.4-dichlorobenzoate, and fluorobenzoate. 

The species identity of the strain was determined as suggested by Chun et al. [28]. The values for ANI and genomic digital DNA–DNA hybridization (dDDH) for strain *Delftia* sp. ULwDis3 (Table 1) with type strain *Delftia tsuruhatensis* NBRC 16741 were 98.6% and 79.3%, respectively, exceeding the values accepted for new species characterization (95% and 70%, respectively) [19,28,29]. The data were used to attribute the strain ULwDis3 to the species *Delftia tsuruhatensis*. We should also note an insignificant difference in DDH and ANI values with respect to the type strains *Delftia tsuruhatensis* NBRC 16741 and *Delftia lacustris* LMG 24775. The obtained results were probably caused by the close evolutionary relationships of the species *Delftia tsuruhatensis* and *Delftia lacustris* within the *Delftia* phylogeny [30].

**Figure 1:**
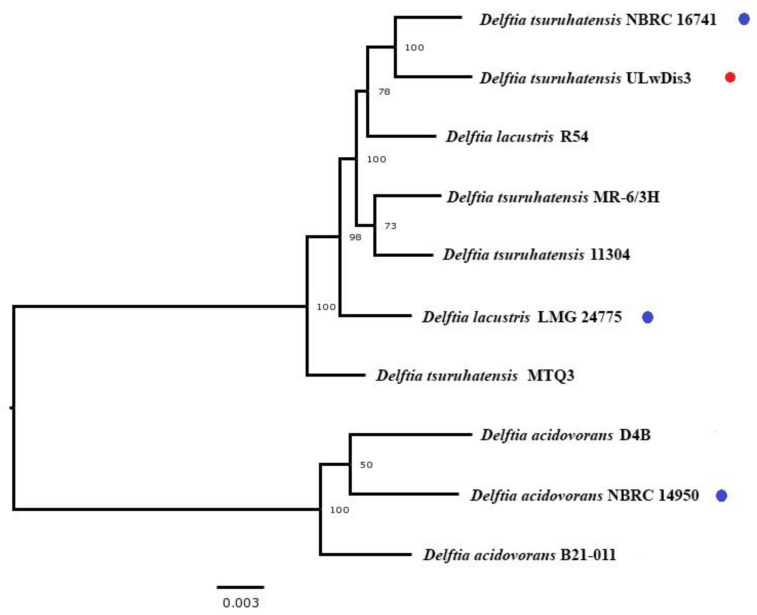
Whole-genome tree of the type *Delftia* strains (blue markers), *Delftia tsuruh*atensis ULwDis3 (red marker) and other members of the species *Delftia* carried out using the PATRIC [20].

The phylogenetic tree was constructed by the neighbor-joining method using the Bacterial and Viral Bioinformatics Resource Center (BV-BRC) database [20]. *Delftia tsuruhatensis* ULwDis3 formed a branch with the type strain *Delftia tsuruhatensis* NBRC 16741, confirming its membership in the species *Delftia tsuruhatensis* (Figure 1). 

The studied strain *D. tsuruhatensis* ULwDis3 was capable of naphthalene degradation. Analysis of genomes of *Delftia* strains available in NCBI databases and on the BV-BRC service did not reveal genome strains possessing genes encoding naphthalene degradation enzymes. To understand the genome structure of *D. tsuruhatensis* ULwDis3, we analyzed the order of genes in the complete genome sequences of the different *Delftia* species strains using the Mauve alignment algorithm [16]. Figure S3 shows the homologous regions of the chromosomes. Nevertheless, we observed a significant difference between the sequence similarity profiles of *D. acidovorans* species and *D. lacustris* and *D. tsuruhatensis* species, which is in agreement with the data of Bhat S.V. et al. [30], who showed that the genus *Delftia* is divided into two well-supported clades: one ‘*Delftia acidovorans*’ clade and a second clade comprising ‘*Delftia lacustris* and *Delftia tsuruhatensis*’. The boundaries of the colored blocks indicate the breakpoints of genomic rearrangements. Some blocks were downshifted relative to others; such blocks were in a reverse complementary (inverse) orientation relative to the studied genome of *D. tsuruhatensis* ULwDis3. It should be noted that common genomic blocks 2000 Kb upstream and downstream of *parA*/*parB* genes were detected for *D. lacustris* and *D. tsuruhatensis* species. The largest genomic rearrangements in the comparison of these two species were observed in the central part of the genome of *D. tsuruhatensis* strains. The locally collinear interconnecting block lines provide an initial indication of the complex rearrangement landscape among the represented related genomes. Several studies have shown that microbial genomes evolve and adapt by integrating new genetic elements through lateral transfer [31,32,33].

### 3.2. Genetic Organization of Catabolic Genes of D. tsuruhatensis Strain ULwDis3

#### 3.2.1. Organization of Naphthalene Degradation Genes (*nag* Genes)

**Figure 2:**
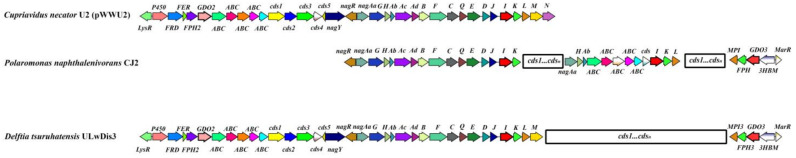
Comparison of the genomic region of the gentisate 1.2-dioxygenase gene in strains *D. tsuruhatensis* ULwDis 3, *P. naphthalenevorans* CJ2, and *C. necator* U2 (pWWU2). *GDO*-gentisate 1.2-dioxygenase, *FPH*–fumarylpyruvate hydrolase, *MPI*–maleylpyruvate isomerase, *3HBM*–3-hydroxybenzoate-6-monooxygenase, *P450*-cytochrome P450, *FRD*-ferredoxin reductase, *FER*–ferredoxin, *ABC*-ABC transporter, *LysR*-LysR-type transcriptional regulators, and *MarR*-MarR-type transcriptional regulators.

The chromosome of the *D. tsuruhatensis* strain ULwDis3 contains all the genes required for the oxidation of naphthalene via salicylate and gentisate to the Krebs cycle intermediates. These genes are organized into a single operon and are located in a region of about 15 kb. In the course of the work, for each gene, its position, G + C content, product size, and proposed function were determined (Table S1, Figure S2). The genetic control of naphthalene degradation via salicylate and gentisate was first described in detail for two members of *β-Proteobacteria*: *Cupriavidus necator* U2 (formerly *Ralstonia* sp.) (ASM966368v2) and *Polaromonas naphthalenivorans* CJ2 (CP000529.1) [34,35]. The structure, DNA sequence, and deduced amino acid sequence of naphthalene catabolic genes on chromosome ULwDis3 were compared with other complete *nag* operons involved in naphthalene degradation (Table S1, Figure S2). The organization and arrangement of the *nag* genes in the ULwDis3 strain are identical to those in the *C. necator* strain U2 [34,36]; however, the naphthalene degradation genes in the latter have plasmid localization (Figure 2). In addition, the *nag* operon of *D. tsuruhatensis* strain ULwDis3 lacks the *nagN* gene encoding a protein with an unknown function. Thus, in the *D. tsuruhatensis* strain ULwDis3, the nag operon contains 17 genes, *nagAaGHAbAcAdBFCQEDJIKLM*. The identity of the deduced amino acid sequences of most *nag* genes of the strain ULwDis3 with the corresponding sequences of the plasmid pWWU2 from *C. necator* strain U2 was 100 % (Table S1). The exceptions were the *nagAd*, *nagB*, *nagF*, and *nagJ* genes, for which the level of amino acid identity was about 99.5–99.8%. The open reading frame (ORF) located upstream of the *nagAa* gene and oriented in the opposite direction from this gene had a high degree of homology (100%) with the corresponding sequence of the naphthalene catabolic strains *C. necator* U2 and *Burkholderia* sp. BC1 (KX155564.1). This sequence encodes the LysR-family transcriptional regulator (NagR) for the expression of the nag operon; NagR has a conserved DNA-binding (helix–turn–helix) domain in the N-terminal region. Upstream of the *nagR* gene is the ORF that putatively encodes the chemotaxis protein, NagY. This ORF is transcribed in the opposite direction from the *nahR* gene. The deduced amino acid sequence of the *nahY* gene also showed a high degree of identity with the corresponding sequences of the strains *C. necator* U2 and *Burkholderia* sp. BC1. The genes of naphthalene degradation via gentisate of *P. naphthalenivorans* strain CJ2 show a significant degree of homology with the nag genes of *C. necator* U2 [35]. However, as in the case of the *D. tsuruhatensis* strain ULwDis3, the catabolic genes of *P. naphthalenivorans* CJ2 have a chromosomal localization. The identities of the deduced amino acid sequences of the nag genes of *D. tsuruhatensis* strain ULwDis3 with the corresponding sequences of strain *P. naphthalenivorans* CJ2 were 31.1–95.9% (Table S1). The lowest similarity in amino acid sequences was observed for fumarylpyruvate hydrolase.

#### 3.2.2. Genes Encoding Gentisate 1.2-Dioxygenases in *D. tsuruhatensis* Strain ULwDis3

Analysis of the genome sequence of the *D. tsuruhatensis* strain ULwDis3 showed the presence of three copies of genes encoding gentisate 1.2-dioxygenase (GDO). One of these genes, *nagI*, is a part of the naphthalene degradation gene cluster (*nag* operon). The gene encoding the second gentisate 1.2-dioxygenase, *GDO2*, is located some distance upstream from the *nag* operon (Figure 2). The identity of the deduced amino acid sequence of this gene with the corresponding sequence of the *nagI* gene of the ULwDis3 strain was only 34.2%. Analysis of the nucleotide sequence of the 5 Kb region upstream of the *GDO2* gene showed that it includes five ORFs. BLAST analysis of the deduced amino acid sequences showed that *GDO2* and at least one ORF (*FPH2*) of this region are involved in the genetic control of gentisate degradation. The four open reading frames of this cluster encoding putative cytochrome P450 (*P450*), ferredoxin reductase (*FRD*), ferredoxin (*FER*), and fumarylpyruvate hydrolase (*FPH2*) are transcribed in the same direction as the *GDO2* gene (Figure 2). The identity of the deduced amino acid sequence of the *FPH2* gene with the corresponding sequence of the nag operon (*nagK*) was no more than 30%. For *FRD* and *FER*, the lack of identity with the deduced amino acid sequences of the *D. tsuruhatensis* strain ULwDis3 *nag*-operon genes was shown. Upstream of the *P450* gene is an ORF that has homology to LysR-type transcriptional regulators. This region, which also includes the *GDO2* gene, had an organization characteristic of bacterial operons. The organization of the abovementioned gene cluster was similar to that of the pWWU2 plasmid (Figure 2). The similarity of the deduced amino acid sequences of the genes included in this cluster in *D. tsuruhatensis* ULwDis3 and *C. necator* U2 (pWWU2) strains was 99–100% (Table S2). Moreover, the region including this gene cluster and the *nag* operon in *D. tsuruhatensis* ULwDis3 and *C. necator* U2 (pWWU2) strains had a similar organization and mutual arrangement of genes. Overall, this region is about 33 Kb (Figure 2).

The third copy of the *D. tsuruhatensis* strain ULwDis3 gentisate 1.2-dioxygenase (*GDO3*) gene had 72.3% similarity of the deduced amino acid sequences with the NagI of the *nag* operon. For the *GDO2* and *GDO3* genes, this value was 34.1%. The *GDO3* gene is part of a cluster whose gene structure and arrangement were similar to the *nagR2ORF2I”KL* gene cluster of *P. naphthalenivorans* CJ2 [35]. In this cluster, *orf2* encodes 3-hydroxybenzoate-6-monooxygenase (3HBM), and the *nagI*, *nagK*, and *nagL* genes encode gentisate 1,2-dioxygenase (GDO3), fumarylpyruvate hydrolase (FPH), and maleylpyruvate isomerase (MPI), respectively. As in the case of *P. naphthalenivorans* CJ2, two ORFs, *FPH3* and *MPI3*, were observed downstream of *GDO3* with amino acid sequence identities of about 79%–99% with fumarylpyruvate hydrolases and maleylpyruvate isomerases from such *Betaproteobacteria* genera as *Delftia*, *Comamonas*, *Variovorax*, etc. (Table S2). Upstream of *GDO3* is an ORF encoding a putative 3-hydroxybenzoate-6-monooxygenase (3HBM) (Figure 2). The identity of the deduced amino acid sequence of *3HBM* with the sequences available in the BLAST database was 78–99% (Table S2). Upstream of the *3HBM* gene, a sequence encoding a putative negative regulator of the MarR-family was found. The *marR* gene is transcribed in the opposite direction from the *3HBM*, *GDO3*, *FPH3*, and *MPI3* genes. The similarity of the deduced amino acid sequences of the abovementioned genes with the genes included in the *nagR2ORF2I”KL* cluster of *P. naphthalenivorans* CJ2 was 50–72% (Table S2). 

The genomes of the genus *Delftia* are characterized by the presence of one or two copies of genes encoding gentisate 1.2-dioxygenase (CP017420.1, AP025556.1, and CP000884.1). Moreover, one of the copies is part of a gene cluster similar to *nagR2ORF2I”KL* of the *P. naphthalenivorans* CJ2 strain. The *D. tsuruhatensis* strain ULwDis3 is the first member of the genus *Delftia* with three sequences encoding gentisate 1.2-dioxygenase found in its genome.

### 3.3. Physiological and Biochemical Characteristics of D. tsuruhatensis Strain ULwDis3 under Cultivation in Liquid Mineral Medium with Naphthalene or Intermediates of Its Degradation Pathway

**Figure 3:**
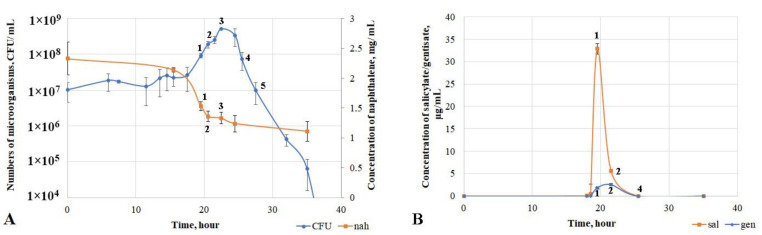
Growth parameters of the *Delftia tsuruhatensis* ULwDis3 strain in a mineral medium with naphthalene (**A**) and accumulation of the key metabolite of the naphthalene degradation pathway (**B**) (nah—naphthalene, sal—salicylate, gen—gentisate).

To assess the key growth parameters of the microorganism, the strain was cultured in mineral medium with naphthalene as the sole source of carbon and energy. The detected growth phases were characterized in terms of metabolite accumulation and evaluation of the activity of key enzymes of the naphthalene degradation pathway. A *lag* phase was observed during the first 12 h of growth of *D. tsuruhatensis* ULwDis3 (Figure 3A). There was no consumption of naphthalene and accumulation of metabolites during this period. There was an increase in the number of bacteria with a simultaneous slight decrease in naphthalene concentration in the medium in the 12–19.5 h growth period. The major process of naphthalene consumption by the culture occurred from 18.5 to 22.5 h (exponential growth phase of the strain), and the degree of naphthalene degradation during this period was 43% (Figure S3).

The activity of the first enzyme of the naphthalene biodegradation pathway, naphthalene 1.2-dioxygenase (NO), was studied (Table 2, Figure S4). The maximum activity of this enzyme was detected at the end of the exponential growth phase (21.5 h). The concentration of naphthalene did not change and remained at 1.25 mg/mL during the period from 25.5 h up to the end of the experiment. At the same time, no NO activity was detected (Table 2, Figure S4). The production and accumulation of the key metabolite of the naphthalene degradation pathway, salicylate, were observed at the point of the exponential start (18.5 h). Accumulation of a small amount of gentisate (up to a concentration of 0.25 μg/mL) was also detected and occurred by 19.5 h of growth. This period was also characterized by a maximum concentration of accumulated salicylate (33 μg/mL) (Figure 3B).

In addition to NO activity, the activities of salicylate 5-hydroxylase (S5H) and gentisate 1.2-dioxygenase (GDO) were measured (Table 2). As a result, maximum S5H activity was observed at the end of the exponential growth phase of the culture. The salicylate concentration had already dramatically decreased to 5 μg/mL at this point (Figure 3B). An increase in GDO activity was observed from 19.5 h of cultivation and peaked by 22.5 h. This indicated the end of the exponential growth phase of the strain. Both metabolites (salicylate and gentisate) were undetectable by the time of 25.5 h of *D. tsuruhatensis* ULwDis3 cultivation with naphthalene as a carbon and energy source. At the same time, the naphthalene concentration did not change, and the growth curve character corresponded to the death phase (Figure 3A).

The NO, S5H, and GDO enzyme activities were also measured in the middle of the exponential growth phase of the strain grown in liquid mineral medium with succinate (Table 2). The NO activity was low during growth of the strain on succinate. This value was comparable with the activity of this enzyme in the middle and end of the exponential growth phase of the strain on naphthalene. S5H activity was absent during growth on succinate. GDO activity values were close to those of GDO under culture growth conditions on naphthalene.

The ability of *D. tsuruhatensis* strain ULwDis3 to utilize salicylate as the sole source of carbon and energy was studied. The strain was cultured in liquid mineral medium with different concentrations of salicylate (0.3, 0.4, 0.5, 0.6, and 0.8 mg/mL). An increase in the adaptation phase of growth was observed depending on the salicylate content in the medium (Table S3). The duration of the exponential growth phase under the conditions of cultivation of the strain with different concentrations of salicylate was about 5 h. 

The growth ability of the culture in liquid mineral medium with 1 mg/mL gentisate as the sole source of carbon and energy was also investigated. The absence of an adaptation period in the *D. tsuruhatensis* strain ULwDis3 was observed, and the duration of the exponential growth phase was 2 h. The initial number of microorganisms was 1 × 10^6^ CFU/mL, and after 2 h, this value reached 1 × 10^8^ CFU/mL.

It is remarkable that when the strain was cultured in liquid mineral medium with naphthalene, there was no stationary growth phase, and the exponential phase was abruptly followed by a dying phase (Figure 3A). Within 48 h after the exponential phase, a stationary growth phase was observed (the number of live cells remained at the level of 10^8^ CFU/mL) when the bacteria were grown in liquid medium with different concentrations of salicylate or gentisate. Thus, there was no rapid death of the culture in the medium with salicylate and gentisate, in contrast to the experiments with naphthalene.

## 4. Discussion

Strains of the genus *Delftia* are *Betaproteobacteria* with agricultural and industrial applications, including plant growth promotion, bioremediation of soils contaminated by hydrocarbons, and immobilization of heavy metals [30]. There is comparatively little information on the degradative properties of these bacteria, as well as on their genome organization. *D. acidovorans* is the most characterized species in the NCBI database. Among 31 genomes of strains of this species, only 8 have been assembled to the complete genome level. Of the 15 genomes of strains of *D. tsuruhatensis* species and 6 genomes of strains of *D. lacustris* species, only 3 complete genome assemblies are available for each. Mauve analysis of different genomes of *D. acidovorans*, *D. tsuruhatensis*, and *D. lacustris* demonstrated the similarity of the genomic profiles of the latter two species. This allowed us to separate the species *D. tsuruhatensis* and *D. lacustris* into a separate common clade, both according to our data and to those reported in [30]. A large number of genomic rearrangements in the central part of the genome were observed in *D. tsuruhatensis* bacteria. This apparently demonstrates a great flexibility of the bacterial genome of this species caused by various transposition events (both within one genome and between genomes of different bacteria). Probably for this reason, the chromosome of the studied strain *D. tsuruhatensis* ULwDis3 contains a 33 Kb region homologous to that of *C. necator* U2.

The naphthalene degraders have not been described to date among *Delftia*, although the ability to grow on different hydrocarbons has been observed in members of this genus. It is assumed that the present work will make some contribution to the understanding of the genetic organization of hydrocarbon degradation genes in strains of the genus *Delftia* and be useful in the direction of studying the biological potential of microorganisms for biodegradation of oil hydrocarbons in contaminated sites. In [6] a strain of the soil bacterium *D. acidovorans* Cs1–4 was found to be capable of degrading phenanthrene, while the strain had no ability to degrade naphthalene. In [7], it was found that the strain *D. lacustris* LZ-C isolated from a petrochemical wastewater discharge site was capable of growth on naphthalene, but the strain lacked the key gene for naphthalene degradation, *nahA*; no data on the degree of hydrocarbon degradation were presented. The *D. tsuruhatensis* strain ULwDis3 was capable not only of growth on naphthalene but also of its degradation (about 40%). In addition to trace amounts of salicylate and gentisate (which degraded over time), no other metabolites were detected during the growth of this strain on naphthalene. Accumulation (but not consumption) of salicylate in a certain period was probably related to cell adaptation to it. This was confirmed by the increase in the *lag* phase in experiments with different concentrations of salicylate. After 25 h of growth on naphthalene, the death of the culture was observed, which was evidently connected not with the accumulation of toxic metabolites but probably with the regulation of catabolic gene expression. This assumption was also confirmed by the absence of a culture death phase during growth with salicylate or gentisate as the sole source of carbon and energy. Salicylate 5-hydroxylase is known to act as a hydroxylating monooxygenase. It is the final link in the chain of electron transport from NAD(F)H to the substrate through ferredoxin reductase (NagAa) and ferredoxin (NagAb). Additionally, ferredoxin mediates both electron transfer to terminal oxygenase (NagAc) and salicylate 5-hydroxylase [25], demonstrating the presence of competition for NAD(F)H between salicylate 5-hydroxylase and naphthalene1.2-dioxygenase, which could theoretically lead to inhibition of the latter and reduction/inhibition of naphthalene utilization [37]. For example, in the present study, we observed a decrease in naphthalene 1.2-dioxygenase activity when growing on naphthalene by the end of the exponential growth phase to a level comparable with this indicator when growing on succinate. The activity of salicylate 5-hydroxylase was still at a high level by the end of the exponential growth phase when growing on naphthalene; when the strain was grown on succinate, salicylate 5-hydroxylase activity was not detected throughout the experiment.

Most of the primary information on the bacterial metabolism of naphthalene (its genetic and biochemical aspects) was obtained on bacteria of the genus *Pseudomonas* of *Gammaproteobacteria* [38,39,40]. For pseudomonads, the most common pathway for naphthalene degradation is its oxidation via catechol; the catabolic genes are organized into two operons [41,42]. The alternative pathway of salicylate utilization via gentisinic acid with the involvement of salicylate 5-hydroxylase in pseudomonads is less common, but the degradation genes are also organized into two operons [43]. The single-operon organization of the genetic pathways of naphthalene degradation via gentisate was first described in detail using the example of *C. necator* U2 [34]. In *D. tsuruhatensis* strain ULwDis3, as well as in the case of plasmid pWWU2 of *C. necator* U2 strain, the *nag* operon is responsible for the degradation of naphthalene to Krebs-cycle intermediates. A similar operon was found in strains of *Burkholderia* sp. BC1 (KX155564.1), *Sphingobium* sp. JS3065 (CP102667.1), and *P. naphthalenivorans* CJ2 (CP000529.1). However, in the latter, the naphthalene catabolic genes are divided into large and small clusters (*nagRAaGHAbAcAdBFCQEDJI*′ORF1tnpA and *nagR2ORF2I “KL*). Initially, *orf2* of the small cluster was thought to encode a new single-component salicylate 5-hydroxylase that does not contain the Riske complex [35]. Later, *orf2* was found to encode a 3-hydroxybenzoate-6-monooxygenase [44]. The similar operon structure of *D. tsuruhatensis* ULwDis3 naphthalene degradation genes and their peculiar arrangement suggest a horizontal transfer of genes from a strain with a similar organization of catabolic pathways with that of the *C. necator* U2 to strain ULwDis3. One confirmation of this is the similar organization of the 33Kb region of the two strains carrying the *nag* operon. Presumably, the presence of IS5/IS1182 and IS256 at the borders of the region is evidence of horizontal transfer of this region from similar strains. Although a similar organization of catabolic naphthalene degradation genes is common in *Betaproteobacteria*, it was described for the first time in this work for bacteria of the genus *Delftia* using the example of *D. tsuruhatensis* strain ULwDis3.

The central pathway for the catabolism of benzoates, phenolic compounds, and PAHs is oxidation through gentisate by gentisate 1.2-dioxygenase [45,46,47,48]. Three sequences encoding gentisate 1.2-dioxygenase were found in the genome of *D. tsuruhatensis* strain ULwDis3. These sequences were included in different gene clusters. No strains carrying three different genes encoding gentisate 1.2-dioxygenase (GDO) were found in the NCBI database among bacteria of the genus *Delftia*. However, there are strains carrying two genes of gentisate 1.2-dioxygenase in their genome (CP065668, DDZR000000000000, and JAEUOW00000000000). It should be noted that in these strains, the genetic organization of one of the catabolic clusters containing *GDO* is similar to the small *nagR2ORF2I “KL* cluster of *P. naphthalenivorans* CJ2. One of the *GDOs* of strain ULwDis3 was also part of a similar cluster. As mentioned above, this gene cluster is responsible for the degradation of 3-hydroxybenzoate and gentisate. Neither the nucleotide nor amino acid composition of the *Delftia* genus strains presented in the NCBI and Patric databases contained sequences similar to the other two GDOs present in the *D. tsuruhatensis* ULwDis3 genome. This also distinguishes this strain from other members of this genus.

The presence of several copies of genes encoding a single enzyme can be physiologically advantageous for the host cell. Due to various regulatory mechanisms, a cell can adjust its metabolism under favorable conditions. When a cell is under conditions of competition for resources (or, in principle, under unfavorable environmental conditions when existing genetic systems contribute minimally to its survival), adaptation can occur in the form of an increase in the number of gene copies or changes in regulatory systems [49,50].

## 5. Conclusions

The conducted studies are important in terms of expanding knowledge of the mechanisms of PAH degradation in bacteria. The identified characteristics of naphthalene degradation by strain *D. tsuruhatensis* ULwDis3 require further transcriptomic and proteomic analyses to determine their regulatory mechanisms. Such analyses can reveal the reasons why naphthalene 1.2-dioxygenase is blocked, preventing complete degradation of all available naphthalene in the system. The latter is important for the development of biotechnological approaches cleaning the environment of oil hydrocarbons by means of degraders.

## Figures and Tables

**Table 1:** ANI and DDH values of *Delftia tsuruhatensis* ULwDis3 with related type strains.

Type Strain	ANI, %	DDH, %
*Delftia tsuruhatensis* NBRC 16741	98.6 *	79.3 *
*Delftia acidovorans* NBRC 14950	95.1	71.6
*Delftia lacustris* LMG 24775	98.33	75.0

* The largest values of ANI and DDH are highlighted in red.

**Table 2:** Activity of the key enzymes of naphthalene degradation.

Substrate	Point	Time, Hours	Enzyme Activity, μmol × mg^−1^ Protein		
			Naphthalene 1.2-Dioxygenase	Salicylate 5-Hydroxylase	Gentisate 1.2-Dioxygenase
**Naphthalene**	1	19.5	11	˂1	32
	2	21.5	49	1148	269
	3	22.5	16	858	2110
	4	25.5	˂1	˂1	251
	5	27.5	˂1	˂1	77
**Succinate**			13	˂1	48

## Data Availability

The data were submitted to the GenBank database under the following accession numbers: BioProject, PRJNA937730; BioSample, SAMN33417407; GenBank, NZ_ CP118775. The strain was deposited in the All-Russian Collection of Microorganisms under the number VKM B-3175D.

## Supplementary Materials

The following supporting information can be downloaded at: https://www.mdpi.com/article/10.3390/microorganisms11041092/s1, Figure S1: Scheme of the naphthalene catabolic pathway via catechol (black arrows) and gentisinic acid (red arrows), Figure S2: Circular map of the *Delftia tsuruhatensis* ULwDis 3 chromosome, Figure S3: Whole-genome comparative chromosome Mauve alignment of different *Delftia* species strains, Figure S4: Dynamics of naphthalene degradation during cultivation of the strain during the experiment, Table S1: Similarity of predicted gene products from the *nag*-gene cluster of *D. tsuruhatensis* ULwDis3 with selected homologs, Table S2: Similarity of predicted gene products from *GDO2* and *GDO3* clusters of *D. tsuruhatensis* ULwDis3 to selected homologs, Table S3: Growth adaptation phase of *Delftia tsuruhatensis* strain ULwDis 3 in liquid mineral medium with different concentrations of salicylate.
