# Supplementary material for: Characterization and Genomic Analysis of the Naphthalene-Degrading Delftia tsuruhatensis ULwDis3 Isolated from Seawater
Source: Microorganisms. 2023 Apr 21;11(4):1092. doi: 10.3390/microorganisms11041092 (PMC10146988; doi:10.3390/microorganisms11041092)
Supplement: Supplementary file 1 [file microorganisms-11-01092-s001.zip › Supplementary material_Microorganisms_Sazonova.pdf]

Supplementary Materials

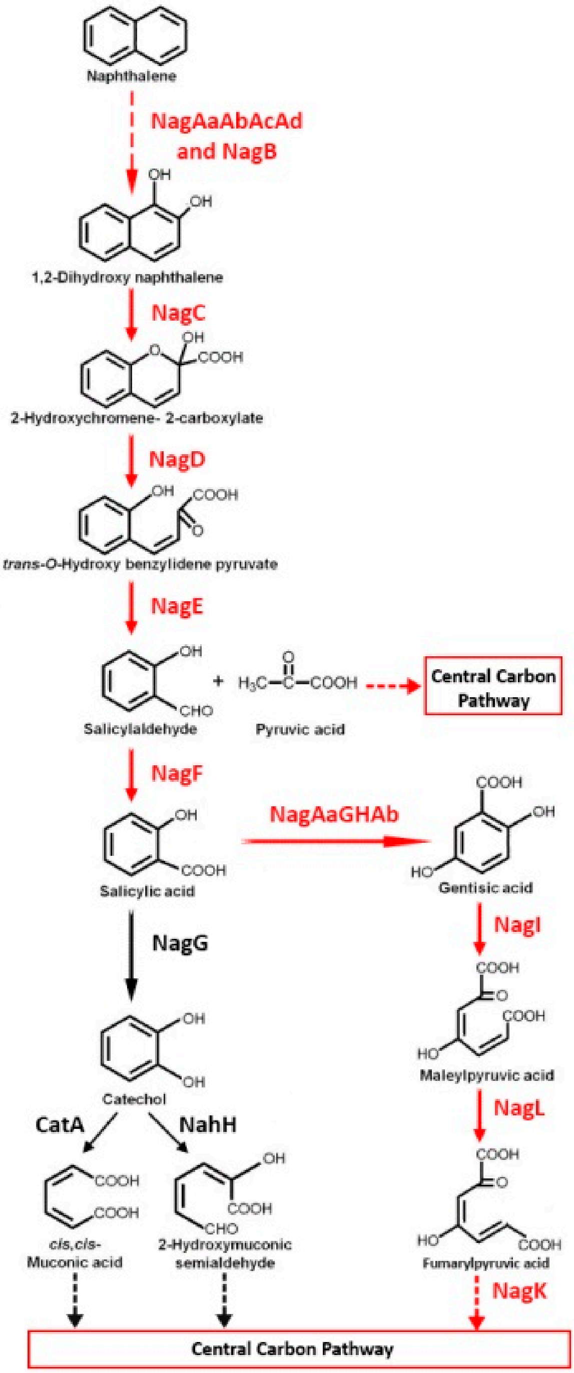

Figure S1. Scheme of naphthalene catabolic pathway via catechol (black arrows) and gentisinic acid (red arrows). The enzymes involved in naphthalene catabolism are naphthalene 1.2-dioxygenase (NagAaAbAcAd), *cis*-naphthalene dihydrodiol dehydrogenase (NagB), 1,2-dihydroxynaphthalene dioxygenase (NagC), 2-hydroxy-2H-chromene-2-carboxylate isomerase (NagD), *trans*-o-hydroxybenzylidenepyruvate hydratase-aldolase (NagE), salicylaldehyde dehydrogenase (NagF), salicylate hydroxylase (NahG), catechol 2,3-dioxygenase (NahH), catechol 1,2-dioxygenase (catA), salicylate 5-hydroxylase (NagGHAaAb), gentisate 1,2-dioxygenase (NagI), maleylpyruvate isomerase (NagL), and fumarylpyruvate hydrolase (NagK). The red color indicates the naphthalene degradation pathway by the *Delftia tsuruhatensis* strain ULwDis3 and the enzymes involved in its catabolism.

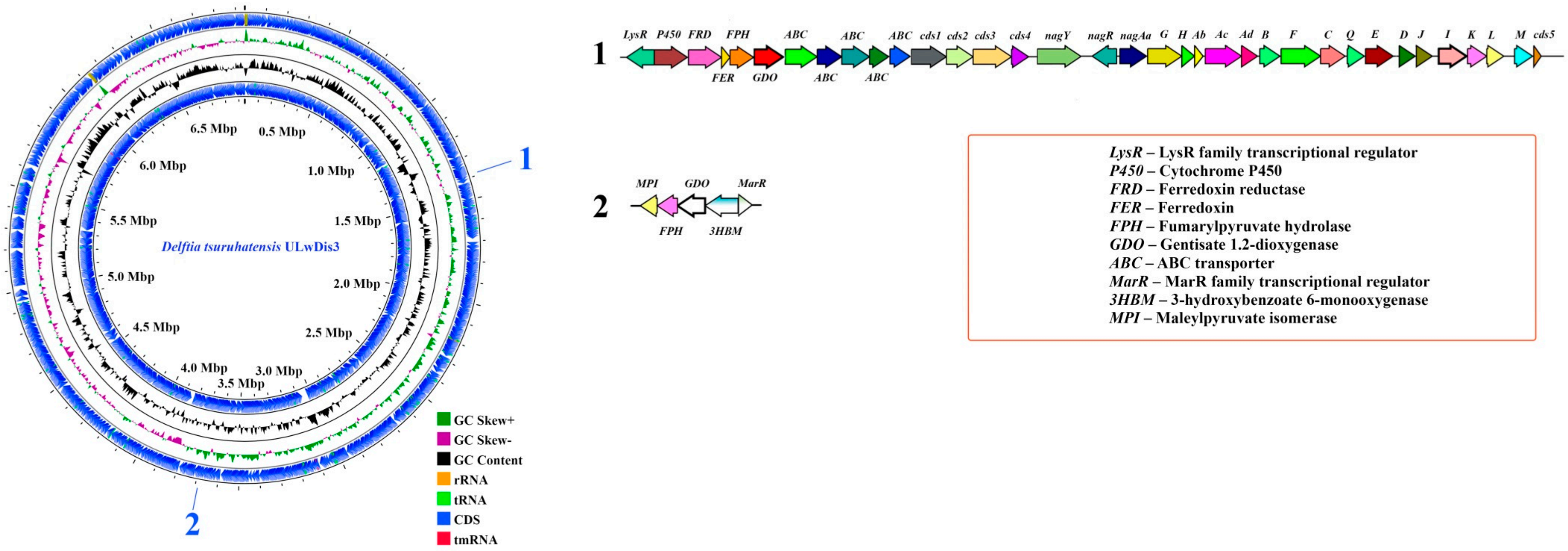

Figure S2. Circular map of the *Delftia tsuruhatensis* ULwDis 3 chromosome. From outside to the center: all CDS and RNA genes on forward strand, GC skew, GC content, and all CDS and RNA genes on reverse strand , .

1 - gene site with two gentisate dioxygenases, including the *nag* operon

2 - gene site with a third gentisate dioxygenase

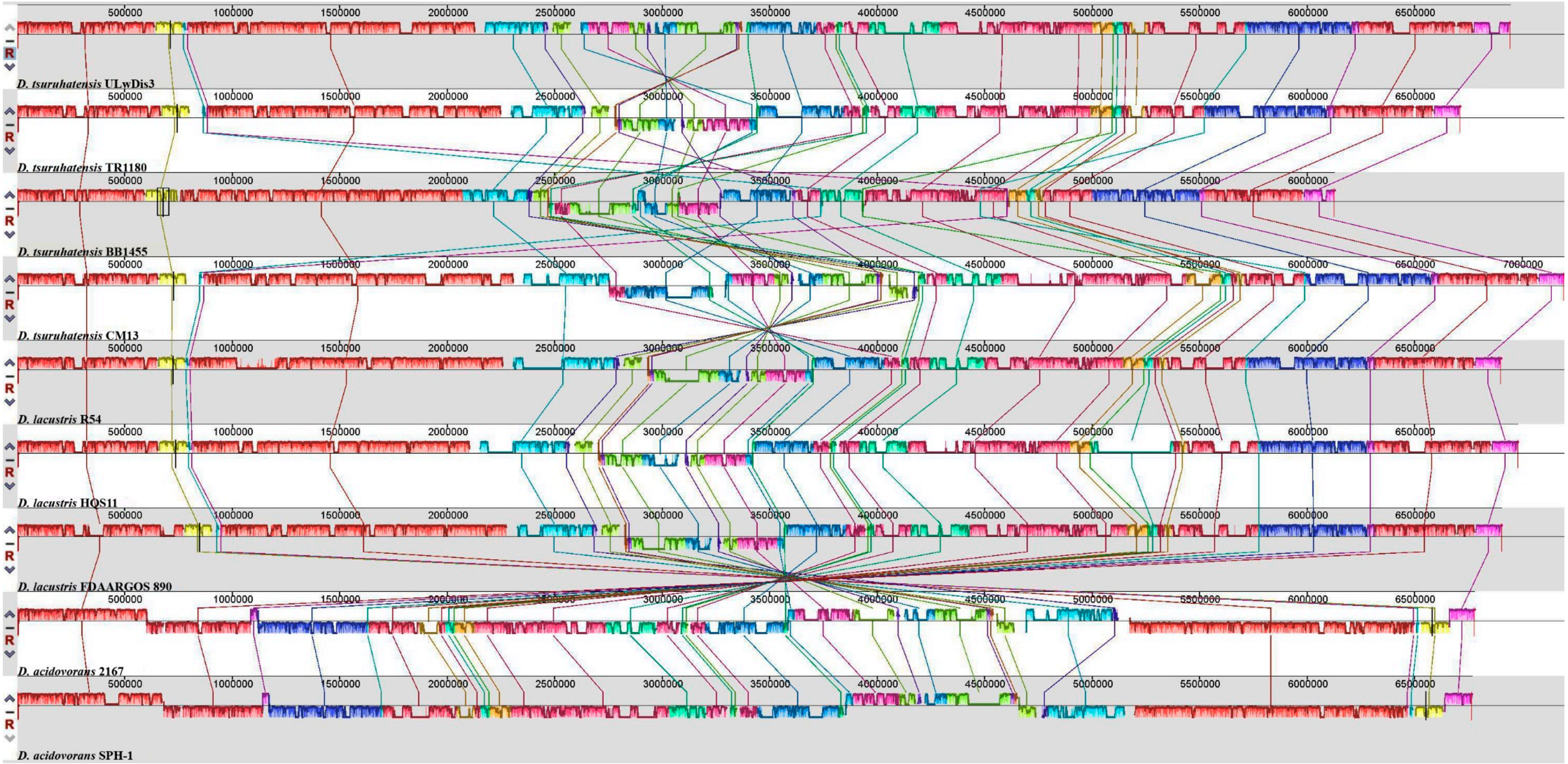

Figure S3. Whole genome comparative chromosomes Mauve alignment of different *Delftia* species strains.

*Colored blocks indicate genome regions aligned with parts of another genome. Each sequence of identically colored blocks represents a collinear set of matching. Homologous blocks in different genomes are connected with lines. Conservation level of genomic regions is shown by the height of the similarity profile.

Table S1. Similarity of predicted gene products from the *nag*-gene cluster of *D. tsuruhatensis* ULwDis3 to selected homologs.

Gene designation	Position (bp)[a]	GC%	Proposed function	No. of amino acids, ULwDis3/ relative[b]	% Amino Acid identity	Source microorganism	GeneBank accession number[c]
nagY	1370733... 1372379d	64.6	Chemotaxis protein	548/548	100	*Cupriavidus necator* U2	KAI3589261.1
				548/429	100	*Burkholderia* sp. BC1	APP18104.1
nagR	1372346... 1373251c	58.4	Transcriptional regulator, LysR family	301/301	100	*Cupriavidus necator* U2	KAI3589262.1
				301/301	100	*Burkholderia* sp. BC1	APP18105
				301/318	83.1	*Polaromonas naphthalenivorans* CJ2	ABM37794.1
nagAa	1373368... 1374354d	61.1	Ferredoxin reductase	328/328	100	*Cupriavidus necator* U2	KAI3589263.1
				328/328	100	*Burkholderia* sp. BC1	APP18106.1
				328/328	75	*Polaromonas naphthalenivorans* CJ2	ABM37793.1
nagG	1374400... 1375671d	61.8	Salicylate-5-hydroxylase, large oxygenase component	423/423	100	*Cupriavidus necator* U2	KAI3589264.1
				423/423	99.1	*Burkholderia* sp. BC1	APP18107.1
				423/420	89.9	*Polaromonas naphthalenivorans* CJ2	ABM37792.1
nagH	1375674... 1376159d	59.5	Salicylate-5-hydroxylase, small oxygenase component	161/161	100	*Cupriavidus necator* U2	KAI3589265.1
				161/161	100	*Burkholderia* sp. BC1	APP18108.1
				161/161	86.3	*Polaromonas naphthalenivorans* CJ2	ABM37791.1
nagAb	1376170... 1376484d	52.4	Ferredoxin	104/104	100	*Cupriavidus necator* U2	KAI3589266.1
				104/104	100	*Burkholderia* sp. BC1	APP18109.1
				104/112	78.8	*Polaromonas naphthalenivorans* CJ2	ABM37790.1
nagAc	1376571... 1377914d	53.2	Naphthalene dioxygenase, large oxygenase component	447/447	100	*Cupriavidus necator* U2	KAI3589267.1
				447/447	98.4	*Burkholderia* sp. BC1	APP18110.1
				447/447	95.3	*Polaromonas naphthalenivorans* CJ2	ABM37789.1

nagAd	1377929... 1378513d	53.7	Naphthalene dioxygenase, small oxygenase component	194/194<br>194/194<br>194/194	99.8<br>94.3<br>89.7	*Cupriavidus necator* U2<br>*Burkholderia* sp. BC1<br>*Polaromonas naphthalenivorans* CJ2	KAI3589268.1<br>APP18111.1<br>ABM37788.1
nagB	1378523... 1379356d	55.9	*cis*-Naphthalene dihydrodiol dehydrogenase	277/273<br>277/259<br>277/259	99.6<br>94.2<br>93.1	*Cupriavidus necator* U2<br>*Burkholderia* sp. BC1<br>*Polaromonas naphthalenivorans* CJ2	KAI3589269.1<br>APP18112.1<br>ABM37789.1
nagF	1379405... 1380856d	59.9	Salicylaldehyde dehydrogenase	483/483<br>483/483<br>483/483	99.6<br>97.1<br>93.6	*Cupriavidus necator* U2<br>*Burkholderia* sp. BC1<br>*Polaromonas naphthalenivorans* CJ2	KAI3589270.1<br>APP18113.1<br>ABM37790.1
nagC	1380882... 1381790d	54.0	1,2-Dihydroxynaphthalene dioxygenase	302/302<br>302/302<br>302/302	100<br>97.7<br>94.1	*Cupriavidus necator* U2<br>*Burkholderia* sp. BC1<br>*Polaromonas naphthalenivorans* CJ2	KAI3589271.1<br>APP18114.1<br>ABM37791.1
nagQ	1381863.. 1382498d	52.9	Outer membrane protein	211/211<br>211/180<br>211/211	100<br>93.2<br>91.5	*Cupriavidus necator* U2<br>*Burkholderia* sp. BC1<br>*Polaromonas naphthalenivorans* CJ2	KAI3589272.1<br>APP18115.1<br>ABM37792.1
nagE	1382556... 1383593d	57.2	*trans-o-*Hydroxybenzylidenepyruvate hydratase–aldolase	345/345<br>345/345<br>345/345	100<br>95.7<br>95.9	*Cupriavidus necator* U2<br>*Burkholderia* sp. BC1<br>*Polaromonas naphthalenivorans* CJ2	KAI3589273.1<br>APP18116.1<br>ABM37793.1
nagD	1383776... 1384375d	51.4	2-Hydroxychromene carboxylate isomerase	199/196<br>199/197<br>199/197	100<br>98.9<br>92.9	*Cupriavidus necator* U2<br>*Burkholderia* sp. BC1<br>*Polaromonas naphthalenivorans* CJ2	KAI3589274.1<br>APP18117.1<br>ABM37794.1
nagJ	1384434... 1385039d	55.4	Glutathione-S-transferase	201/201<br>201/201	99.5<br>99.5	*Cupriavidus necator* U2<br>*Burkholderia* sp. BC1	KAI3589275.1<br>APP18118.1

				201/201	93.5	*Polaromonas naphthalenivorans* CJ2	ABM37795.1
nagI	1385270...1386310d	67.1	Gentisate 1,2-dioxygenase	346/346	100	*Cupriavidus necator* U2	KAI3589276.1
				346/355	100	*Burkholderia* sp. BC1	APP18119.1
				346/334	85.5	*Polaromonas naphthalenivorans* CJ2	ABM37796.1
nagK	1386344...1387045d	64.0	Fumarylpyru-vate hydrolase	233/233	100	*Cupriavidus necator* U2	KAI3589277.1
				233/192	100	*Burkholderia* sp. BC1	APP18120.1
				233/289	31.1	*Polaromonas naphthalenivorans* CJ2	ABM37797.1
nagL	1387057...1387695d	67.3	Maleylpyruvate isomerase	212/212	100	*Cupriavidus necator* U2	KAI3589278.1
				212/212	100	*Burkholderia* sp. BC1	APP18121.1
nagM	1387791...1388783d	59.2	Unknown	330/282	100	*Cupriavidus necator* U2	KAI3589279.1
				330/341	100	*Burkholderia* sp. BC1	APP18122.1

[a] Letters indicate coding strand: c, complementary strand; d, direct strand.

[b] Number of amino acids in ULwDis3 chromosome CDS/number in the closest relative.

[c] GenBank accession number of the closest relative protein.

Table S2. Similarity of predicted gene products from *GDO2* and *GDO3* clusters of *D. tsuruhatensis* ULwDis3 to selected homologs.

Gene designation	Position (bp)[a]	GC%	Proposed function	No. of amino acids, ULwDis3/ relative[b]	% Amino Acid identity	Source microorganism	GeneBank accession number[c]
				GDO2 cluster			
LysR	1355489.. 1356406c	55.9	Transcriptional regulator, LysR family	305/305	100	*Cupriavidus necator* U2	KAI3589245.1
				305/305	100	*Comamonas testosteroni* YAZ2	BCX54896.1
				305/339	100	*Burkholderia multivorans* BC00014	MBJ9658604.1
P450	1356541.. 1357779d	62.4	Biotin biosynthesis cytochrome P450	412/412	99.8	*Cupriavidus necator* U2	KAI3589246.1
				412/412	100	*Comamonas testosteroni* YAZ2	BCX54897.1
				412/412	100	*Burkholderia multivorans* DDS 15A-1	AIO76613.1
FRD	1357787.. 1359025d	65.7	Ferredoxin reductase	412/412	100	*Cupriavidus necator* U2	KAI3589247.1
				412/396	99.8	*Xenophilus azovorans* DSM 13620	WP_211260262.1
				412/412	100	*Comamonas testosteroni* YAZ2	BCX54898.1
FER	1359044.. 1359364d	58.9	Ferredoxin	105/106	100	*Cupriavidus necator* U2	KAI3589248.1
				105/105	100	*Comamonas testosteroni* YAZ2	BCX54899.1
FPH2	1359375.. 1360244d	62.5	Fumarylpyru-vate hydrolase	289/289	100	*Cupriavidus necator* U2	KAI3589249.1
				289/286	75.1	*Variovorax paradoxus*	QFZ85153.1
				289/289	92.4	*P. naphthalenivorans* CJ2	ABM37779.1
GDO2	1360241.. 1361317d	62.5	Gentisate 1,2-dioxygenase	358/358	100	*Cupriavidus necator* U2	KAI3589250.1
				358/328	99.7	*Comamonas thiooxydans* JC8	NZ_AWOS01000062
				358/351	78.5	*Paraburkholderia phytofirmans* OLGA172	ANB74996.1
				GDO3 cluster			
MPI3	3685200.. 3685841c	70.7	Maleylpyruva-te isomerase	213/213	99.5	*Delftia* sp. HK171	TQL81322.1
				213/213	79.8	*Variovorax* sp. HW608	SCK13974.1

				213/214	87.7	*Comamonas* sp. AG1104	RDI10688.1
				213/212	72.3	*P. naphthalenivorans* CJ2	ABM38445.1
FPH3	3685861.. 3686568c	65.9	Fumarylpyruvate hydrolase	235/235	89.7	*Acidovorax* sp. ST3	WP_110958765.1
				235/245	100	*Delftia lacustris* HQS1	BDE71824.1
				235/235	88.9	*Comamonas* sp. Tr-654	NIF82459.1
				235/235	82.1	*P. naphthalenivorans* CJ2	ABM38444.1
GDO3	3686607.. 3687644c	70.4	Gentisate 1,2-dioxygenase	345/345	84.1	*Acidovorax* sp. CCYZU-2555	MBS7781544.1
				345/345	99.1	*Delftia* sp. CH05	MXN31744.1
				345/351	73.9	*P. naphthalenivorans* CJ2	ABM38443.1
3HBM	3687661.. 3688869c	69.5	3-hydroxybenzoate 6-hydroxylase	402/402	99.6	*Delftia* sp. CH05	MXN31745.1
				402/402	91.1	*Acidovorax* sp. KKS102	AFU46209.1
				402/402	99.5	*Delftia tsuruhatensis* TR1180	QFS65584.1
				402/400	78.6	*P. naphthalenivorans* CJ2	ABM38442.1
marR	3689074.. 3689610d	69.3	Transcriptional regulator, MarR family	178/178	97.1	*Delftia acidovorans* FDAARGOS_909	QPS09337.1
				178/182	80.3	*Comamonas* sp. AG1104	RDI10685.1
				178/178	98.9	*Delftia tsuruhatensis* TR1180	QFS65583.1
				178/161	50.4	*P. naphthalenivorans* CJ2	ABM38441.1

[a] Letters indicate coding strand: c, complementary strand; d, direct strand.
[b] Number of amino acids in ULwDis3 chromosome CDS/number in the closest relative.
[c] GenBank accession number of the closest relative protein.

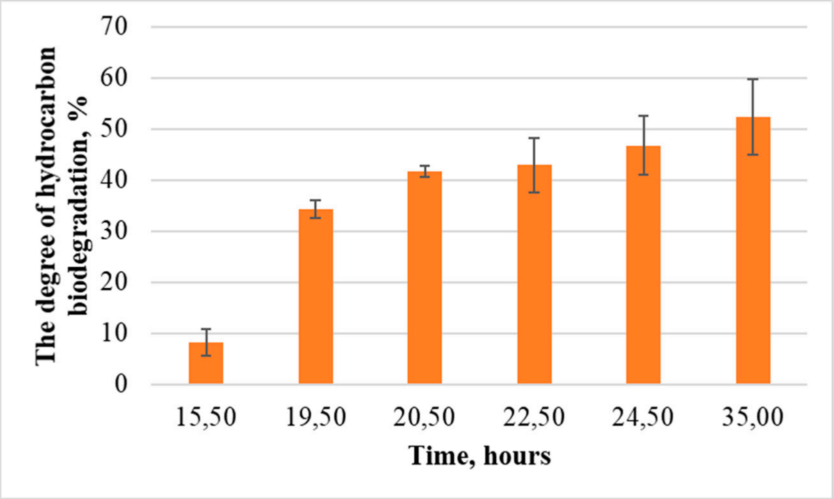

Figure S4. Dynamics of naphthalene degradation during cultivation of the strain during the experiment

Table S3. Growth adaptation phase of *Delftia tsuruhatensis* strain ULwDis 3 in liquid mineral medium with different concentrations of salicylate

Salicylate concentration, g/l	Adaptation growth phase, hours
0,3	23
0,4	40
0,5	55
0,6	80
0,8	174
